# A Paradox in Digital Memory Assessment: Increased Sensitivity With Reduced Difficulty

**DOI:** 10.3389/fdgth.2021.780303

**Published:** 2021-11-22

**Authors:** Joshua P. White, Adrian Schembri, Chris J. Edgar, Yen Ying Lim, Colin L. Masters, Paul Maruff

**Affiliations:** ^1^Cogstate Ltd, Melbourne, VIC, Australia; ^2^Cogstate Ltd, London, United Kingdom; ^3^School of Psychological Sciences, Turner Institute for Brain and Mental Health, Monash University, Clayton, VIC, Australia; ^4^Florey Institute of Neuroscience and Mental Health, The University of Melbourne, Melbourne, VIC, Australia

**Keywords:** cognition, digital biomarker, memory, Alzheimer's, diagnosis

## Abstract

The One Card Learning Test (OCL80) from the Cogstate Brief Battery—a digital cognitive test used both in-person and remotely in clinical trials and in healthcare contexts to inform health decisions—has shown high sensitivity to changes in memory in early Alzheimer's disease (AD). However, recent studies suggest that OCL sensitivity to memory impairment in symptomatic AD is not as strong as that for other standardized assessments of memory. This study aimed to improve the sensitivity of the OCL80 to AD-related memory impairment by reducing the test difficultly (i.e., OCL48). Experiment 1 showed performance in healthy adults improved on the OCL48 while the pattern separation operations that constrain performance on the OCL80 were retained. Experiment 2 showed repeated administration of the OCL48 at short retest intervals did not induce ceiling or practice effects. Experiment 3 showed that the sensitivity of the OCL48 to AD-related memory impairment (Glass's Δ = 3.11) was much greater than the sensitivity of the OCL80 (Glass's Δ = 1.94). Experiment 4 used data from a large group of cognitively normal older adults to calibrate performance scores between the OCL80 and OCL48 using equipercentile equating. Together these results showed the OCL48 to be a valid and reliable test of learning with greater sensitivity to memory impairment in AD than the OCL80.

## Introduction

The importance of digital technology to decision-making in the field of the neuropsychology of Alzheimer's disease (AD) is growing rapidly (1–3). As both pre-symptomatic and symptomatic AD are characterized most strongly by difficulty with learning and memory, digital tools have been applied to many aspects of the assessment of these functions. For example, digital tools have been developed to provide scoring algorithms and reports for data collected using conventional paper and pencil cognitive tests (4), to replace the written and graphical material used as the stimuli that constrain the written or spoken responses of patients (5, 6), and also to measure the speed of manual responses on such tests (3). While the common application of digital tools in AD neuropsychological settings has been to replace components of standardized assessment or reporting, other approaches have sought to exploit digital technologies so they do not simply recapitulate the printed-spoken-written approaches of clinical interviews. For example, assessments of learning and memory have been designed specifically for computer administration, making use of the precision timing for presentation of stimuli and recording of responses. Such tests also make use of computer software to analyze in real time, performance data in order to adjust instructional and training procedures so individuals understand and adhere to the rules and requirements of the tests (7, 8). Such refinement has allowed one of these digital tools, the Cogstate Brief Battery (CBB), to extend aspects of neuropsychological decision-making from one-on-one clinical interviews (9, 10), to supervised settings in large groups (11), high frequency repeated testing (12, 13) and pre-interview triage (14), as well as wide-scale unsupervised internet-based cognitive assessment (15–17). Despite this success, it remains important to continue the refinement and optimization of digital tools, especially where use cases identify the need for such improvement. Understanding such cases, as well as the methods and solutions developed for improvement, should contribute to the knowledge base concerning the optimal design characteristics for novel digital assessments tools for application in AD contexts.

**Figure 1:**
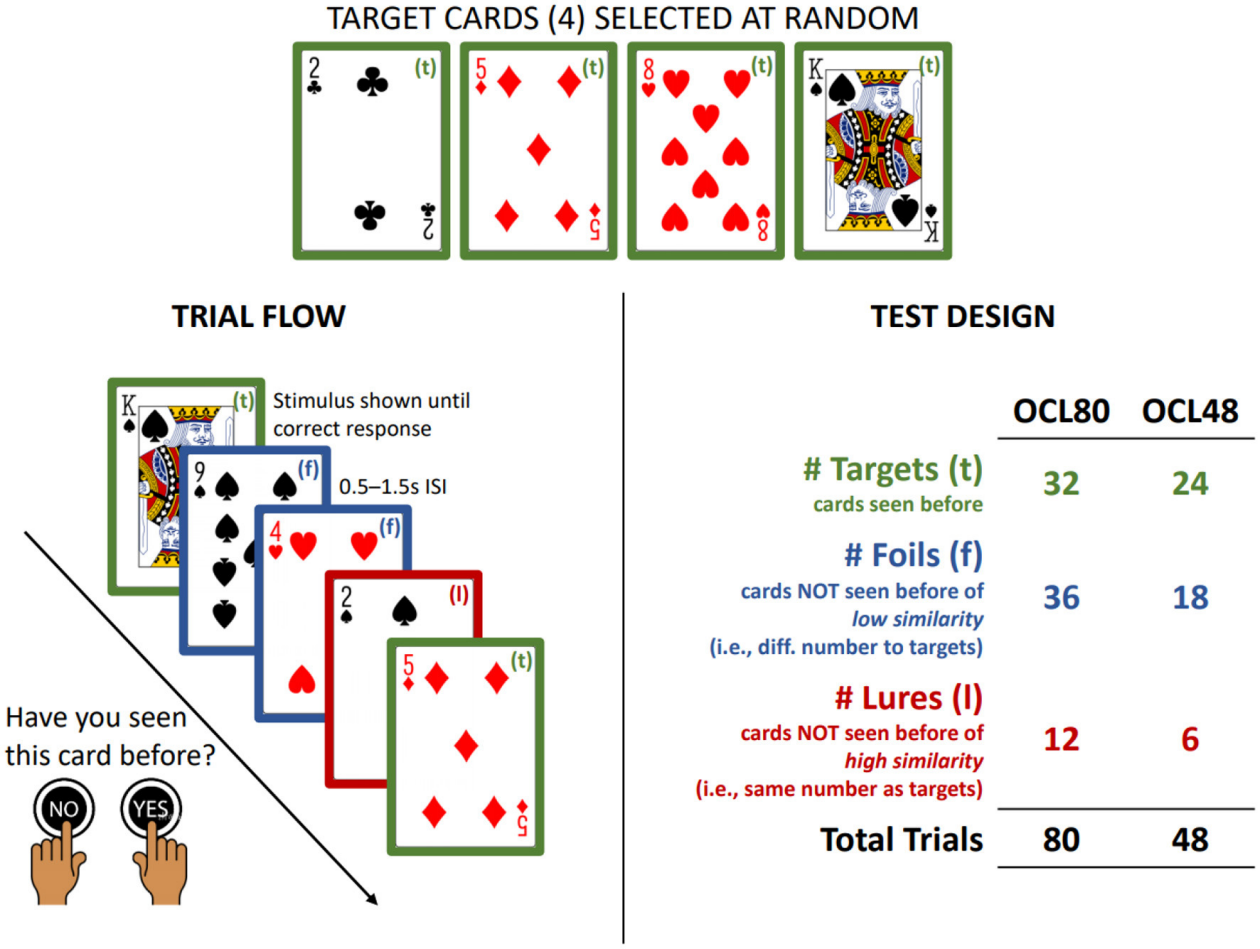
Summary of OCL48 and OCL80. Summary of the trial flow and design of the OCL48 and OCL80. ISI, Inter-Stimulus Interval.

The test of visual learning from the CBB is the One Card Learning test (OCL, Figure 1). The OCL has been shown to be sensitive to the cognitive deterioration that characterizes symptomatic and pre-symptomatic Alzheimer's Disease (18, 19) as well as to the effects of experimental drugs designed to minimize memory deficits in AD (20). As early pathological changes in AD occur in medial temporal lobe areas, the sensitivity of the OCL to memory decline in this disease stage may reflect its design being based on the pattern separation model of memory (21, 22). Pattern separation memory models contend that medial temporal lobe areas are necessary for the development of memory engrams that allow organisms to discriminate among highly similar and unique pieces of information (e.g., different faces in a crowd) (21, 23). The more similar to-be-learned information is to information already stored in memory, the more difficult it is for the organism to classify it as novel (24) and the ability to discriminate new from old information in a flow of highly similar information is reduced by disruption to processing in the medial temporal lobe (21, 23, 25). As playing cards have overlapping visual features, discrimination of new from old information in a stream of 80 cards most likely renders the OCL sensitive to such disruption. Furthermore, similarity between playing card stimuli can be increased further by organizing the information stream so that some remembered and novel cards share multiple values with targets (e.g., number and color).

The sensitivity of the OCL to AD-related memory decline has resulted in extension of its use to clinical settings where it is intended to guide decisions about the presence of cognitive impairment in individuals at risk for AD (14, 26, 27). One important difference between decisions about cognitive decline and cognitive impairment, is that while decisions about cognitive decline are based on comparison of test performance within-individuals over time, decisions about cognitive impairment are based on comparison of performance to reference data from cognitively normal (CN) older adults. The results from some studies using the OCL in AD show its sensitivity to memory impairment to be lower than expected for some classifications (27, 28). Consideration of data from these cross-sectional AD studies indicates that performance in this group was often close to chance (i.e., 50%) suggesting there is a floor effect in test performance in these groups. This floor effect could have restricted the range of scores between the mean OCL performance in CN adults and symptomatic AD, so, even at their worse, OCL performance in symptomatic AD could only be 1.5 standard deviations below that of the CN adults (27, 28). Thus, optimization of the OCL for use in decisions about memory change may not generalize to its appropriateness for application to decisions about memory impairment. In addition to reducing the floor effect for memory impairment, the sensitivity of the OCL to abnormal memory in symptomatic AD may also be improved if average performance in CN groups was also better. In 60–70-year-old adults, average performance on the standard OCL is 1.0 (arcsine square root proportion correct) with a standard deviation of 0.10 (26). Thus, there exists potential to increase average performance on the OCL in normal groups, as the maximum possible score for this test, 1.57 (26), is ~4 standard deviation units above the average score. Thus, a lower than optimal performance in CN adults and performance floor effects in symptomatic AD could both be overcome through a reduction in the difficulty of the OCL. This raises the hypothesis, paradoxical in many neuropsychological contexts and in the design of digital cognitive tests generally, that to improve the sensitivity of the OCL to AD-related cognitive impairment it is necessary to make it easier.

The OCL used in AD studies to date has contained 80 trials (hereafter termed the OCL80). The design of this is summarized in Figure 1. Participants see one playing card at a time in the center of the visual display and must decide “yes” or “no” whether they had seen that card previously. On each administration of the OCL80, trials are presented in which some cards are targets and others are distractors. Distractors are divided again on the basis of their similarity to the targets, with 12 sharing the numerical value with the target (lures) and the 24 selected at random from the remaining cards (foils). To reduce the difficulty of the OCL to improve its sensitivity to AD-related memory impairment, a shorter 48-trial version of the OCL was created, called the OCL48 or the reduced difficulty OCL. To ensure its similarity to the longer OCL80, the ratio of lures to foils in each administration remained unchanged. Thus, in the OCL48, only four targets were presented six times (*n* = 24), and the number of distractors reduced to 24, six of which were lures and 18 were foils.

An experimental psychology approach was utilized to guide the optimization of the OCL48. Four experiments were conducted to test the hypothesis that reducing the difficulty of the OCL would improve its sensitivity to AD-related memory impairment while retaining its validity, stability, and reliability. Investigation of one test characteristic at a time in individual experiments using highly controlled samples increases the precision of measurement and thereby allows for use of smaller samples than when all characteristics of interest are examined in a single study. Therefore, the first experiment examined the validity of the reduced-difficulty OCL by determining whether in CN adults, it retained its dependence on memory operations consistent with pattern separation models. The second experiment determined the extent to which the reduced difficulty OCL might give rise to practice effects with repeated application at short retest intervals in CN adults. The third experiment investigated the sensitivity of the conventional and reduced difficulty OCL to AD related memory impairment. The fourth experiment developed a mathematical framework for calibrating performance on the conventional and reduced difficulty OCL. The methods and results of the experiments are reported in sequence to illustrate the assumptions made and steps taken to optimize the sensitivity of the OCL to AD-related memory impairment.

## Experiment 1: Simplification of the OCL and Pattern Separation Memory

Pattern separation models of recognition memory and learning show that individuals have much greater difficulty rejecting information that is highly similar to that stored in memory (24) and that this is increased in older adults (24, 29). This study investigated in CN adults whether with reduced difficulty, performance on the OCL would improve while retaining its dependence on pattern separation memory operations. To do so, we utilized a mixed factorial experimental design, in which an older and a younger group of CN participants completed both the OCL48 and OCL80, allowing us to examine whether trial-by-trial performance differed according to the OCL difficulty or the age of the participants.

### Methods

#### Participants

Participants were 56 cognitively normal adults who had completed both versions of the CBB as part of their participation in other studies. Twenty-one CN normal young adults (CN younger group) were recruited from the general population using a word-of-mouth method while 35 CN older participants (CN older group) were recruited from the Australian Imaging, Biomarker & Lifestyle (AIBL) study. The methods for screening, recruitment, and assessment of participants in the AIBL study has been detailed previously (30). For the CN younger group, the requirement for inclusion was that they self-reported no current or history of neurological disorder and that they were generally in good health. Each participant in this group identified themselves as being physically healthy and absent of intercurrent neurological or psychiatric disease. The CN older group underwent detailed neuropsychological test battery and medical workup as part of the AIBL study (30). Only individuals without chronic systemic disease, cancer or cerebrovascular disease were included. Cognitively normality in the CN older group was determined by an expert consensus panel that examined all available cognitive, neurological, and psychiatric information for each participant. The demographic characteristics of the CN older group and CN younger group are shown in Table 1.

**Table 1:** Demographic and Neuropsychological characteristics of CN old and CN younger groups.

	**CN younger group**	**CN older group**
*N*	21	35
**Sex**		
Male *N* (%)	11 (59.1%)	10 (28.57%)
Female *N* (%)	10 (40.9%)	25 (71.43%)
**Age**		
*M* (*SD*)	35.38 (6.11)	79.94 (5.80)
Range	22–44	73–98
**Education**		
<13 Years *N* (%)	10 (47.62%)	0 (0%)
13–15 Years *N* (%)	11 (52.38%)	20 (57.14%)
>15 Years *N* (%)	0 (0%)	15 (42.86%)
**MMSE**		
*M* (*SD*)	–	29.83 (0.45)
Range	–	28–30
**FSIQ**		
*M* (*SD*)	106.05 (4.46)	91.24 (8.70)
Range	100–115	72.55–112.3
**HADS depression**		
*M* (*SD*)	1.62 (0.86)	2.26 (1.96)
Range	1–4	0–9

*CN, Cognitively normal; MMSE, Mini-Mental State Examination; FSIQ, Full-Scale IQ; HADS depression, Hospital Anxiety and Depression Scale – depression subscale*.

#### Measures

##### OCL80

For both OCL difficulty versions, participants sat facing a computer screen on a desk. A single large button was placed each side of the computer screen, and participants were trained by the computer software to push the right-hand side button to answer “yes” and the left-hand button to answer “no.” No other engagement with the computer or the computer keyboard was required of participants. The OCL was presented on the computer monitor. The OCL48 and OCL80 have the same design in which a single card is presented face down in the center of the screen. The participant is trained so that when the central card flips, they must respond as quickly as they can by pressing either the “yes” or “no” button in response to the rule; posed as the question “Have you seen this card before?” If the response is correct, the card flips to the bottom of the pack to reveal the next card, starting the next trial making the next response necessary. If the participant answers incorrectly, the card remains at the central location and the participant must select the other (correct) option. For example, the correct answer to the first card shown in the test is always “no” (as, by definition, the very first card has not previously been seen). If a participant presses the right-hand button (i.e., respond “yes”), then the card remains present until they make the correct response (i.e., “no,” the left-hand button). This process continues until the participant has seen each of the cards scheduled to be presented in the test.

The OCL80 presents four playing cards, selected at random from the standard 52-card deck, as targets with the restriction that one must be from each suit. In a single administration, each target card is shown eight times, yielding a total of 32 targets in a test. Once presented, each target card is shown only again after the other three target cards have been presented, although the order in which target cards are shown is random within these groups. The correct response for each target card is “yes.” The 48 playing cards from the deck not defined as targets are then organized with the following constraints. For each target, the three cards that have the same numerical value are selected and defined as lures. For example, if the two of hearts card is selected as a target, then the two of diamonds, two of clubs and two of spades are defined as lures. This yields 12 lures being presented in each test administration. The remaining 36 cards are selected at random from the remaining cards in the deck and are defined as foils. Each lure and foil card is presented only once in the test, in random order, and interspersed pseudo-randomly with the target cards such so that within each cycle of presentation of the four target cards, six distractor cards (lures or foils) are presented, yielding a total of 80 trials. The correct response for all distractor cards is “no.”

##### OCL48

The OCL48 is identical to the OCL80 in visual display, rules for performance and response requirements. As stated above the memory demands of the OCL48 were reduced by reducing the ratio of distractor to target cards. The test was also shortened by removing two cycles of target presentations (and the associated presentation of distractors) to improve acceptability. The OCL48 utilizes four target cards, and 24 non-target cards selected and presented as for the OCL80. The OCL48 differed from the OCL80 in that target cards were presented six times (rather than eight) yielding 24 targets in a test. The second difference was that 24 non-target cards were presented in a single administration of which 6 were classified as lures and 18 as foils. Within each cycle of presentation of the four target cards, there occur four distractor cards presented once in the test, in pseudorandom order. This yielded a total of 48 trials. Figure 1 summarizes the trial flow and test design of both the OCL48 and OCL80.

#### Procedure

All individuals completed the OCL80 and OCL48 on different days with the order of administration of the two tests randomized at the first assessment. Each test was completed by each participant once only. The computer software noted the version of the test administered so that the non-selected version was presented automatically on retesting. Participants were not told which version of the OCL they performed. Participants were introduced to the computer, trained on using the “yes” and “no” buttons and then began the test under the supervision of a rater. The rater read the standard instructions and commenced administration of the test until completion.

#### Data Analysis

The data files from each administration of the OCL48 and OCL80 were inspected by computer statistical software to compute the proportion of correct responses made across all trials. The statistical programs then aggregated data to express the number of correct responses made to targets, foils and distractor trials on each assessment, arcsine square root transformed. The pattern separation indices were then investigated by submitting the total correct responses to lures, foils, and targets to a 3 (trial type: lure, foil, or target) × 2 (test difficulty: OCL48 vs. OCL80) × 2 (Age group: younger vs. older) mixed ANOVA, to ascertain whether trial-by-trial performance on the OCL differed according to any of these experimental treatments. The Greenhouse-Geisser sphericity correction was used when Mauchly's test indicated that the sphericity assumption was violated. Significant interactions were decomposed using *t*-tests. Where variances between groups were not equal Welch's *t-*test degrees of freedom approximation was used. To minimize the potential for Type I error, the levels of significance required for interpretation was *p* < 0.01. In addition, standardized measures of effect size (Cohen's d) were also computed for all comparisons of interest to provide estimates of the magnitude of experimental effects.

### Results and Discussion

The 3 × 2 × 2 mixed ANOVA showed a significant main effect for test difficulty, *F*_(1, 54)_ = 10.46, *p* = 0.002. A paired samples *t*-test indicated that mean accuracy in the OCL48 (*M* = 1.09, *SD* = 0.12) was significantly greater than mean accuracy in the OCL80 (*M* = 1.00, *SD* = 0.10), *t*_(55)_ = 5.08, *p* < 0.001, *d* = 0.68. There was also a main effect for trial type, *F*_(1.67, 90.04)_ = 192.87, *p* < 0.001. *Post-hoc* paired *t*-tests showed that mean accuracy (averaged over OCL versions) was significantly greater for foil trials (*M* = 1.02, *SD* = 0.13) than for lure trials (*M* = 0.68, *SD* = 0.22), *t*_(55)_ = 13.89, *p* < 0.001, *d* = 1.86; and for target trials (*M* = 1.24, *SD* = 0.15) compared to foil trials, *t*_(55)_ = 8.17, *p* < 0.001, *d* = 1.50.

**Figure 2:**
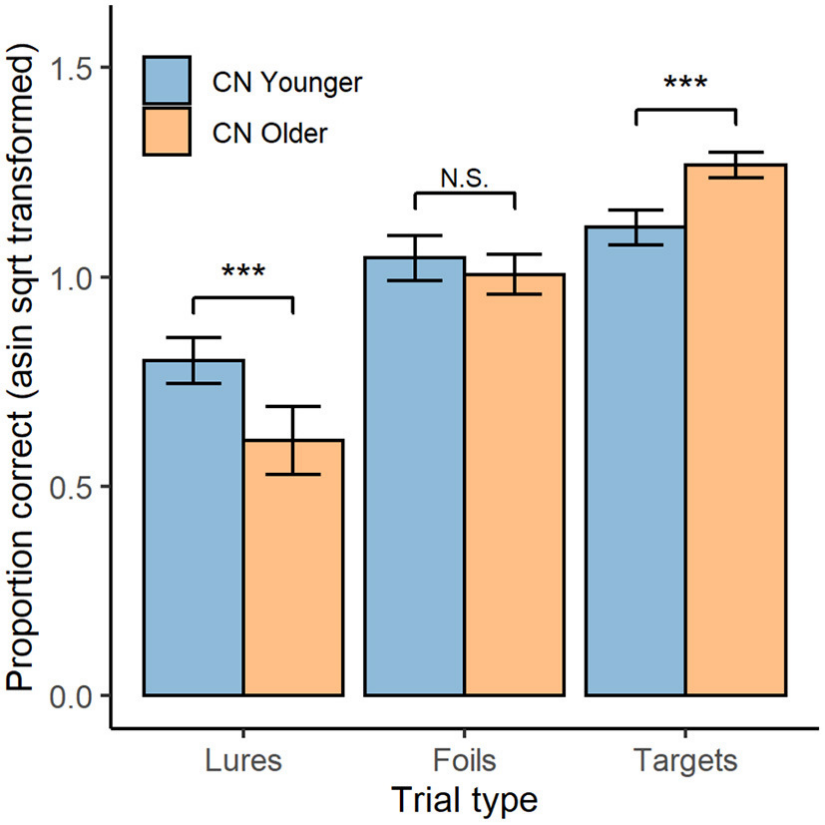
OCL Performance by trial type and age group. Mean performance on both the OCL48 and OCL80 for each trial type (lures, foils, and targets; x-axis), for both younger (blue) and older (orange) groups. Error bars represent 95% confidence intervals. Asin sqrt, arcsine square root N.S., non-significant. ***p < 0.001.

The highest order, and only, significant interaction in the ANOVA was the age group × trial type interaction; *F*_(1.67, 90.04)_ = 27.06, *p* < 0.001 (shown in Figure 2). The interaction was decomposed by comparing age groups for each trial type with independent-samples *t*-tests (averaged over OCL versions). For lure trials, accuracy was higher in the younger (*M* = 0.80, *SD* = 0.13) than the older group (*M* = 0.61, *SD* = 0.24), *t*_(53.22)_ = 3.86, *p* < 0.001, *d* = 0.99. For foil trials, accuracy was equivalent in the younger (*M* = 1.05, *SD* = 0.13) and older (*M* = 1.01, *SD* = 0.14) groups, *t*_(54)_ = 1.04, *p* = 0.303. For target trials, accuracy was lower in the younger (*M* = 1.31, *SD* = 0.13) than the older (*M* = 1.12, *SD* = 0.10) group, *t*_(55)_ = 5.71, *p* < 0.001; *d* = 1.58.

These data indicated that reducing the difficulty of the OCL led to a substantial improvement in group mean performance with raw scores improving by 0.09 units resulting in a standardized effect size for that improvement of 0.67. Despite the reduction in difficulty, within-individual differences in performance between the lure, foil, and target trials on the OCL80 and OCL48 indicated that the memory operations necessary for performance remained constrained by pattern separation memory operations for both versions of the test. This is shown by performance on lure trials being less accurate than performance on foil trials (*d* = 1.86), which was in turn less accurate than performance on target trials (*d* = 1.50). It is now important to ensure that the reduction in difficulty of the OCL does not lead to ceiling or practice effects.

## Experiment 2: Stability oF the OCL48 Over Brief Retest Intervals in Healthy Young Adults

One consequence of a reduced difficulty OCL may be that individuals with normal memory function could obtain perfect scores (i.e., ceiling effects), or show substantial improvements in performance with repeated administration (practice effects). Because the optimal condition for use of the OCL to guide decisions about cognitive change requires there be no practice effects associated with re-testing, and because psychological models of learning show that benefits to performance arising from repeated exposure to the same cognitive challenges occur most strongly over initial trials, as described by the learning curve (31–33), this study challenged the stability of the reduced difficulty OCL by repeating assessment on the same people three times at short retest intervals.

### Methods

#### Participants

Participants were 23 healthy young adults (12 females, 10 males, 1 = NA, *M*_age_ = 22.57, *SD* = 3.58, range: 19–30) who completed the CBB repeatedly. All participants were university students who self-reported that their cognition was normal. All participants gave informed consent to undergo the CBB.

#### Measures

The OCL48 used in this study was described for Experiment 1.

#### Procedure

Participants attended a laboratory at the university where they remained and completed the OCL48 three times at 2-hour intervals.

#### Data Analysis

Data processing was described in Experiment 1. Group mean proportion correct scores were compared between timepoints with repeated measures MANOVA profile analysis using the *paos* function from the R package *profileR* (34), to ascertain whether mean performance differed at any timepoint. To determine if ceiling or floor effects occurred for individuals in the study, the proportion of participants that scored the maximum score (1.57) or below a chance-level score (0.79) were also computed for each assessment. A floor or ceiling effect was classified to exist if more than 10% of participants score at these levels (35). Finally, estimates of reliability and within subject standard deviation were also computed.

### Results and Discussion

Table 2 shows the accuracy of performance of the OCL48 over 3 re-administrations, each 2-hour apart. Repeated measures MANOVA profile analysis showed no difference in accuracy across timepoints; Hotelling's *T*^2^ = 0.50, *F*_(2, 20)_ = 0.24, *p* = 0.79. The intraclass correlation was 0.63, 95% CI [0.40, 0.81]; *F*_(21, 63)_ = 6.94, *p* < 0.001. Within-subject standard deviations ranged from 0.01 to 0.15, with the mean within-subject standard deviation being 0.06. No participant obtained a perfect score (1.57) or a score at or below chance level ( ≤ 0.79) at any timepoint.

**Table 2:** OCL48 performance over repeated measures.

	**Baseline**	**Hour 2**	**Hour 4**	**WSD**
Mean (*SD*)	1.1 (0.1)	1.08 (0.14)	1.09 (0.1)	0.06 (0.03)
Range	0.83–1.32	0.81–1.43	0.93–1.32	0.01–0.15
*N* max scores (i.e., 1.57)	0	0	0	-
*N* min scores (i.e., ≤ 0.79)	0	0	0	-

*WSD, Within-subject standard deviation*.

Despite the reduced difficulty of the OCL48, there was no evidence of ceiling or practice effects in healthy young adults assessed repeatedly over short retest intervals. This indicates that the OCL48 remains appropriate for use in studies designed to measure change despite its reduced difficulty. The potential for OCL48 to improve sensitivity of AD-related memory impairment can now be investigated.

## Experiment 3: Sensitivity of the OCL48 to Memory Impairment in AD

To determine whether a reduction in difficulty would improve sensitivity of the OCL to AD-related memory impairment, the magnitude of difference in performance between an AD dementia group and matched CN older adults, both with disease status confirmed by amyloid biomarker levels, was compared between the OCL48 and OCL80.

### Methods

#### Participants

Participants were 52 adults who completed the CBB as part of their participation in the AIBL study (30), and had undergone positron emission tomography (PET) neuroimaging using Pittsburgh Compound-B (PiB) to determine levels of beta-amyloid (Aβ). The process for recruitment, screening and assessment of individuals in the AIBL study has also been described in detail elsewhere (30). Participants were grouped according to clinical diagnosis and Aβ level. A total of 22 participants were classified as CN older adults with Aβ levels within normal limits (i.e., CN Aβ− group), and a total of 30 participants were classified as having AD dementia and abnormally high Aβ (i.e., AD dementia; AD Aβ+ group). Participants were classified as Aβ+ if their PiB-PET standardized uptake value ratio was ≥1.4 (36, 37). Cognitive normality of participants was determined by an expert clinical panel who reviewed all neuropsychological, psychiatric, and medical information, but who were blind to participants' Aβ status. Similarly, AD dementia was classified by the same consensus panel according to the NINCDS-ADRDA criteria (38). Table 3 contains the demographic and clinical information for both groups.

**Table 3:** Demographic, Neuropsychological, and Biomarker characteristics of CN Aβ− and AD Aβ+ groups.

	**CN Aβ− Group**	**AD Aβ+ Group**
*N*	22	30
**Sex**		
Male *N* (%)	13 (59.1%)	11 (36.7%)
Female *N* (%)	9 (40.9%)	19 (63.3%)
**Age**		
*M* (*SD*)	79.41 (4.53)	80.60 (4.43)
Range	73–86	70–87
**MMSE**		
*M* (*SD*)	29.36 (1.00)	18.55 (5.15)
Range	27–30	10–28
**FSIQ**		
*M* (*SD*)	108.05 (7.66)	101.11 (8.85)
Range	87–118	86–115
**CDR SOB**		
*M* (*SD*)	0.09 (0.33)	5.57 (3.12)
Range	0–1.5	1–17
**HADS depression**		
*M* (*SD*)	2.43 (1.95)	5.30 (3.75)
Range	0–6	1–19
**PET PiB SUVR**		
*M* (*SD*)	1.12 (0.06)	2.24 (0.31)
Range	1.02–1.23	1.73–3.11

*MMSE, Mini-Mental State Examination; FSIQ, Full-Scale IQ; CDR SOB, Clinical Dementia Rating Sum of Box; HADS depression, Hospital Anxiety and Depression Scale – depression subscale; PET PiB SUVR, Positron Emission Tomography Pittsburgh Compound-B Standardised Uptake Value Ratio*.

#### Measures

The OCL48 and OCL80 were administered to participants exactly as described above for experiment 1.

#### Procedure

All participants completed the OCL80 and OCL48 within the same testing session with the two tests given in last position in the CBB. The order was randomized so that some performed the OCL80 first, and others the OCL48 first. All participants had completed the CBB (and as such, the OCL) on previous occasions. Instructions for the OCL80 and OCL48 were the same.

#### Data Analysis

Data processing was described in Experiment 1. Type III, two-way mixed ANOVA was used to ascertain whether accuracy differed as a function of test difficulty (OCL48 vs. OCL80), diagnosis (CN Aβ− v AD Aβ+) and their interaction. Statistically significant effects were decomposed using *t*-tests and standardized measures of effect size were also computed to allow for the appreciation of the magnitude of experimental effects. To compare the magnitude of impairment associated with AD between the OCL version Glass's Δ was used as an effect size for magnitude of difference on each test between the CN Aβ− and AD Aβ+ groups. Cohen's d was used as a standardized effect size for comparisons between performance on the OCL48 and OCL80 test.

### Results and Discussion

**Figure 3:**
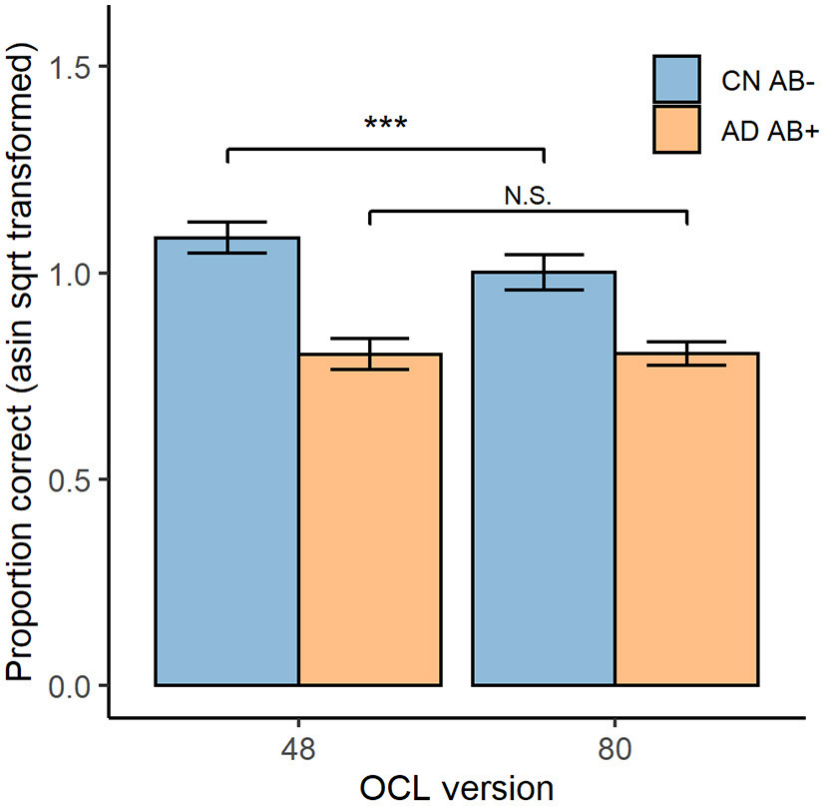
OCL48 and OCL80 Performance for CN Aβ− and AD Aβ+ participants. Overall performance on both the OCL48 and OCL80 tests for both cognitively normal beta-amyloid negative (CN Aβ−; blue) and Alzheimer's Diseased beta-amyloid positive (AD Aβ+; orange) groups. Error bars represent 95% confidence intervals of the means. Asin sqrt, arcsine square root N.S., non-significant. ***p < 0.001.

Figure 3 displays test accuracy grouped by diagnosis group and OCL test version. ANOVA indicated a significant diagnosis × difficulty interaction, *F*_(1, 50)_ = 7.76, *p* < 0.001. The paired sample *t*-tests indicated that the CN Aβ− group showed significantly higher accuracy on the OCL48 (*M* = 1.09, *SD* = 0.09) than on the OCL80 (*M* = 1.00, *SD* = 0.10), *t*_(21)_ = 4.84, *p* < 0.001, *d* = 1.03. However, for the AD Aβ+ group, performance did not differ significantly differ between the OCL48 (*M* = 0.80, *SD* = 0.11) and OCL80 (*M* = 0.80, *SD* = 0.08); *t*_(29)_ = −0.07, *p* = 0.947, *d* = 0.01.

Comparison of the sensitivity of each OCL to AD dementia related memory impairment showed the effect size for impairment in the AD group was greater for the OCL48 (Glass's Δ = 3.11, 95% CI [2.00, 4.21]) than for the OCL80 (Glass's Δ = 1.94, 95% CI [1.16, 2.69]) with regression modeling showing this difference to be significant; unstandardized interaction effect = 0.09, 95% CI [0.01, 0.16], *t*_(100)_ = 2.289, *p* = 0.024.

These data support the hypothesis that reducing the difficulty of the OCL while retaining its theoretical and psychometric characteristics would improve the sensitivity of the test to AD-related memory impairment.

## Experiment 4: Equating Scores Between the OCL48 AND OCL80

Given the strong theoretical and operational consistency between the OCL48 and OCL80 and the greater sensitivity of the OCL48 to AD-related memory impairment, it is important to understand the relationship between performance on the two OCL versions so as to provide a framework for their direct comparison. We therefore calibrated scores on the OCL80 and OCL48 using equipercentile equating (39). Equipercentile equating was used as it produces equating estimates that allow for test difficulty to scale differently across the range of each test, and thus allows for a non-linear relationship between the tests (39).

### Methods

#### Participants

Participants were 401 healthy adults from the United States of America recruited via Amazon Mturk (249 female, 152 male, *M*_age_ = 68.12, *SD* = 6.03, age range: 55–80).

#### Measures

The OCL48 and OCL80 were administered to participants exactly as described above for experiment 1.

#### Procedure

After being recruited to participate via Amazon Mturk, participants completed some basic demographic criteria, before completing both the OCL48 and the OCL80. The order that participants took the tests was randomized between participants to control for order effects.

#### Data Analysis

Equipercentile equating was conducted using the *equate* package in R (40). Prior to analysis, scores from either test <0.79 were assigned the value 0.79 because this score indicates at-chance performance; the lower bound for the OCL tests. This ensured that equating the distributions would begin at chance performance level and would not result in listwise deletion of scores less than chance. Prior to equating, loglinear pre-smoothing was completed for both test distributions to avoid overfitting to the present dataset in the equating process (40), using the default setting from the *equate* package. Data was equated at 0.01 intervals from chance (0.79) to maximum (1.57) for each test. Finally, because equipercentile involves the estimation of many quantiles for each distribution, results can be affected by sampling variability in these estimates, particularly at distributional extremes (41). A non-parametric bootstrapping approach was thus conducted to ascertain the standard error for each equated score. Finally, this process was repeated in the reverse direction, to also equate scores on the OCL80 to scores on the OCL48.

### Results and Discussion

In this sample, average performance was superior for the OCL48 (*M* = 1.07, *SD* = 0.11, min = 0.79, max = 1.57) than the OCL80 (*M* = 0.99, *SD* = 0.10, min = 0.79, max = 1.32), *t*_(400)_ = 13.45, *p* < 0.001, *d* = 0.67. Table 4 contains the equated scores on the OCL80 from the OCL48 for all scores of the OCL48 and bootstrapped estimates of the standard error. Supplementary Table 1 contains the full equating analysis undertaken at every 0.01 interval between 0.79 (minimum) and 1.57 (maximum), both equating from the OCL48 to OCL80, and vice versa from the OCL80 to OCL48.

**Table 4:** OCL48 scores and their OCL80 equivalent, estimated with equipercentile equating.

***N* correct**	**OCL48 score**	**OCL80 equivalent**	**SE**
24/48	0.79	0.79	0.0009
25/48	0.81	0.79	0.0038
26/48	0.83	0.80	0.0059
27/48	0.85	0.81	0.0074
28/48	0.87	0.82	0.0086
29/48	0.89	0.83	0.0095
30/48	0.91	0.84	0.0101
31/48	0.93	0.85	0.0105
32/48	0.96	0.88	0.0105
33/48	0.98	0.90	0.0103
34/48	1.00	0.92	0.0101
35/48	1.02	0.94	0.0098
36/48	1.05	0.97	0.0094
37/48	1.07	0.99	0.0091
38/48	1.10	1.02	0.0086
39/48	1.12	1.04	0.0085
40/48	1.15	1.07	0.0086
41/48	1.18	1.10	0.0092
42/48	1.21	1.13	0.0104
43/48	1.24	1.15	0.0120
44/48	1.28	1.19	0.0147
45/48	1.32	1.21	0.0184
46/48	1.37	1.24	0.0253
47/48	1.43	1.26	0.0467
48/48	1.57	1.45	0.1005

*SE refers to bootstrapped standard error. OCL48 scores are arcsine square root transformed*.

The mean standard error of the equating was 0.04, and the *weighted* mean standard error of the equating was 0.01. Analysis of the bootstrapped standard errors for each value showed that equating error was generally low (0.01 or less) for most scores on the OCL48 distribution up to where correct responses occur on 47 or more of the 48 responses (Table 4) with error increasing at these levels. However, under optimal conditions, ceiling effects are uncommon on this test (Experiment 2) and therefore data shown in Table 4 provide a reliable basis for calibration of most performance on the OCL48 to that on the OCL80.

## Conclusion

The OCL is a digital tool that has been used extensively to measure change in memory but for which required further optimization to improve its sensitivity to memory impairment in AD. This study therefore sought to optimize the sensitivity of the OCL to memory impairment in AD using an experimental psychological methodology. The results of the four experiments conducted indicated that reducing the difficulty of the OCL improved its sensitivity to AD-related memory impairment when compared to that of the original version (i.e., OCL80). These results also indicate two important principles for the design and optimization of digital tools for use in neuropsychological contexts. First, that a difficult memory test is not always the most sensitive test of memory, and second that in optimizing digital neuropsychological tools it is important to consider separately sensitivity to impairment and sensitivity to change. Despite the reduced difficulty, the OCL48 retained its dependence on the memory operations described by pattern separation models of memory (21, 22, 24) and did not give rise to ceiling or practice effects when applied repeatedly under optimal conditions. The consistency in the test design, theoretical context, and performance characteristics indicated that the OCL48 could be used effectively in studies of AD while continuing to be constrained by the body of research that has guided the development and interpretation of the OCL paradigm (18, 19, 42). With these factors operating, it became possible to develop a meaningful and accurate calibration process for equating performance scores between the two levels of OCL test difficulty.

While the standard OCL80 had been shown in many settings to be sensitive to changes in memory (18–20), recent studies applying this to characterize memory impairment in individuals classified clinically with mild cognitive impairment (MCI) or with biomarker positive status, showed lower than expected binary classification accuracy (although equivalent to that of delayed auditory verbal learning), did not discriminate between biomarker subgroups within cognitively unimpaired individuals and showed poor sensitivity using conventional cut-offs (27, 28). *Post-hoc* analysis of the outcomes from these studies suggested that the lower-than-expected sensitivity arose from a restriction in the range of the performance scores. This arose because performance on the OCL80 in the individuals with AD dementia was typically at or close to floor (i.e., chance), while there remained the substantial potential for performance in cognitively normal adults to have higher values. A reduction in test difficulty would result in improved performance (higher group mean scores) in cognitively normal adults and in adults with memory impairment, thereby reducing the potential for floor effects and increasing the range of scores between dementia and cognitively normal groups. This line of reasoning led to the seemingly paradoxical path, at least in neuropsychology, where the sensitivity of the OCL to memory impairment would be increased through having its difficulty reduced. The experiments in this study show that this was the case.

The difficulty of the conventional OCL was reduced by limiting the number of times each target card was presented (from 8 to 6) and then reducing the number of distractor cards accordingly, while retaining the ratio of lures and foils (3 to 1). This modification reduced the number of trials on the OCL from 80 to 48, which also made the test time for administration substantially shorter by ~1 min. Experiment 1 demonstrated that this reduction in difficulty did not disrupt the relationship between OCL performance and the pattern separation memory operations, with both younger and older individuals showing lower accuracy when rejecting high similarity lures than when rejecting the lower similarity foils (Figure 2) (24). Experiment 2 showed that reducing the difficulty of the OCL did not lead to an opposite problem of biasing performance toward ceiling effects, even in healthy young adults with normal memory performance. With these characteristics established, the cardinal experiment in this study showed that the less difficult OCL48 had greater sensitivity to AD-related memory impairment. Inspection of effect sizes for differences in OCL performance between AD and normative groups indicated that for the OCL48, impairment (3.11 SDs) was more than a standard deviation greater than that observed for the OCL80 (1.94 SDs). The experimental nature of this study and the tight inclusion criteria used to define both the cognitively normal and AD groups mean that the differences observed here may not generalize to clinical settings. However, as a proof of principle, the reduction in difficulty on the OCL changed the magnitude of memory impairment in AD from less than two standard deviations below normal levels, to greater than three standard deviations below normal levels. If replicated in clinical contexts, the reduction in the difficulty of the OCL should result in an improvement in its sensitivity and specificity of identifying memory impairment due to AD.

Analysis of the performance issues that had reduced the sensitivity of the standard OCL (OCL80) to AD-related memory impairment, led to the hypothesis that a reduction in OCL difficulty should improve performance in AD groups. Interestingly, inspection of the estimates of group mean performance in Experiment 3 indicated that while cognitively normal older adults did show better performance on the OCL48 (group mean ~1.09) than on the OCL80 (group mean ~1.00), there was not a substantial improvement in performance with the OCL48 in the AD group (Figure 3). Thus, the improved sensitivity of the OCL48 occurred because accuracy of performance increased in the cognitively normal older adults. For the AD group, reduction in the difficulty of the OCL (i.e., the OCL48) did not influence accuracy of performance substantially with group mean performance levels remaining near chance performance (Figure 3). The relative absence of the effects of OCL difficulty on performance in AD dementia has two consequences for use of the OCL48 in clinical contexts. First, it suggests that the OCL48 will have limited utility for distinguishing mild from moderate AD dementia. However, this will not limit the use of the OCL in clinical settings as this test is used mostly to assist in confirming objectively the presence of memory impairment in individuals whose clinical cognitive status is unclear, rather than classifying dementia severity (26, 43). There currently exist many well-validated instruments (e.g., MMSE, MOCA) that can accurately characterize disease severity in individuals diagnosed with dementia, which can be used in conjunction with the OCL48. The second issue arising from the larger range between performance in the cognitively normal older adults and the adults with AD dementia on the OCL48 is that this increased range of scores between group mean normal and abnormal performance may increase the sensitivity of the OCL to the memory impairment that characterizes the AD prodrome, or MCI (44). It is now necessary to repeat Experiment 3 in adults with MCI due to AD to understand whether test difficulty can influence performance in these individuals.

The results of this study also suggest that pattern separation models of memory may provide a strong theoretical context for understanding memory impairment in AD, as well as a basis for building strong brain behavior models of the genesis of this dysfunction (29, 45, 46). Pattern separation models of memory are useful in that they are constrained by sophisticated computational structures, animal lesion and behavioral models and human cognitive neuroimaging and behavioral studies (22). It is often challenging to translate human cognitive models into disease groups because the tasks typically are very long and complex in terms of their perceptual, motor or decision requirements. The results from the OCL48 shown here suggest that in additional to its acceptability in the context of clinical diagnosis and clinical trials, this test may provide a useful tool for bridging outcomes to the sophisticated brain-behavior models of pattern separation.

The results of this study also contribute to the literature on the development and use of digital healthcare tools in the diagnosis and management of AD. First, the study illustrates how it is possible to develop interactive digital tools that despite their novelty remain constrained by modern brain behavior models of memory and neuropsychological models of brain disease (1, 46). The results show a process for the identification and optimization of dimensions of performance of digital tools in their intended context of use that utilizes knowledge from experimental psychology, statistics and computer technology and shows a pathway for test optimization conducted using a series of highly controlled experiments (3). Finally, this study illustrates how optimization of a digital tool for one use in making specific decisions in a target group, may not generalize when the same tool is used in the same group to make different decisions about the same aspect of cognition.

Some important caveats to the current findings must be considered when seeking to generalize these to other contexts. First, each was an experiment where the study sample and method for test administration was highly controlled. Consequently, while the findings of an increased sensitivity to memory impairment in AD with the OCL48 are reassuring, formal assessment of data in a clinical context is required before claims about sensitivity or specificity can be made. Second, although the sample used for the calibration (equating) of performance between the OCL48 and OCL80 was large, there remained some larger than expected error associated with estimates of performance at the very high end of the performance scales (i.e., where only one or two errors are made). This is most likely due to these estimates being based on small proportions (i.e., that proportion at the 95th and greater percentiles) of the normative sample. Therefore, a larger sample is required to obtain a calibration algorithm for all cases that require translation of the score from the OCL48 to OCL80 or vice versa, likely with a larger sample size to allow for more precise estimation of quantiles at the upper extreme of the score distribution. Nonetheless, the accuracy of prediction for scores derived when more than two errors per test were made—most of the performance even for cognitively normal groups—all had very low error, indicating that score equating can be applied with greater confidence for such values.

Future work now must evaluate the OCL48 as it pertains to the other tests in the Cogstate Brief Battery. In particular, various composite scores are calculated from the multiple tests in the CBB (e.g., Learning and Working Memory composite), and they must now be evaluated for performance when used with the OCL48 rather than the OCL80. Indeed, it is possible that composites other than those currently in use for the CBB may be improved and found useful including the OCL48 rather than the OCL80. In addition, future studies are needed to evaluate the use of the OCL48 in clinical settings.

## Conclusion

The present study analyzed the psychometric properties of a reduced-length, reduced-difficulty version of the Cogstate OCL test: the OCL48. The results confirm that the OCL48 is both easier and more sensitive than the standard OCL (OCL80) to memory impairment in AD, a counterintuitive result in neuropsychological contexts in which, generally, increased sensitivity comes with increased difficulty. Furthermore, the OCL48 retained its pattern separation structure, stability, and resistance to ceiling and practice effects even after close-in-time repeated administrations. The OCL48 may thus be used in lieu of the OCL80 as it retains desirable psychometric properties, has greater sensitivity, and is shorter to administer.

## Data Availability Statement

The raw data supporting the conclusions of this article will be made available by the authors, without undue reservation.

## Ethics Statement

Exp 1, Exp 3 was approved by the St Vincent's Human Research and Ethics Committee, St Vincent's Hospital Melbourne, Australia. Exp 2, Exp 4 was approved by Monash University Research and Ethics Committee, Monash University, Melbourne Australia. The patients/participants provided their written informed consent to participate in this study.

## Author Contributions

JW and PM conceptualized, wrote, and revised the paper. JW undertook statistical analyses and generated data visualizations. AS and PM conceptualized and designed the OCL48 presented in this study. PM and CE supervised the project. All authors read and approved the submitted version.

## Funding

The AIBL study, from which data for experiments 1 and 3 from this paper was obtained, (www.AIBL.csiro.au) received support from Commonwealth Scientific and Industrial Research Organisation, the Science and Industry Endowment Fund (www.SIEF.org.au), National Health and Medical Research Council, and Dementia Collaborative Research Centres. YL was supported by an NHMRC Career Development Fellowship (GNT1162645). Experiment 2 was conducted using data collected with partial funding from Cogstate Ltd. Experiments 1, 3, and 4 were conducted on data derived from studies that had received no funding from Cogstate Ltd, although employees of Cogstate (JW, AS, CE, and PM), did participate in the data analyses, interpretation of data and in the writing of the manuscript. In Experiment 3, employees of Cogstate (JW, AS, CE, and PM) participated in study conceptualization and design, data analyses, interpretation of data and in the writing of the manuscript although they were not involved in recruitment or study conduct.

## Conflict of Interest

JW, AS, CE, and PM are/were full time employees of Cogstate Ltd, the company that distributed the Cogstate Brief Battery. The remaining authors declare that the research was conducted in the absence of any commercial or financial relationships that could be construed as a potential conflict of interest.

## Publisher's Note

All claims expressed in this article are solely those of the authors and do not necessarily represent those of their affiliated organizations, or those of the publisher, the editors and the reviewers. Any product that may be evaluated in this article, or claim that may be made by its manufacturer, is not guaranteed or endorsed by the publisher.
