# Supplementary material for: A Paradox in Digital Memory Assessment: Increased Sensitivity With Reduced Difficulty
Source: Front Digit Health. 2021 Nov 22;3:780303. doi: 10.3389/fdgth.2021.780303 (PMC8645569; doi:10.3389/fdgth.2021.780303)
Supplement: Supplementary file 1 [file Table_1.DOCX]

**Supplementary Material**

**Table S1.** Equipercentile equating analysis undertaken at every 0.01 interval between 0.79 (minimum) and 1.57 (maximum), both equating from the OCL48 to OCL80, and vice versa from the OCL80 to OCL48.

| Equating OCL80 scores from OCL48 | | | Equating OCL48 scores from OCL80 | | |
| --- | --- | --- | --- | --- | --- |
| OCL48 score | OCL80 equivalent | SE | OCL80 score | OCL48 equivalent | SE |
| 0.79 | 0.79 | 0.0009 | 0.79 | 0.80 | 0.0066 |
| 0.80 | 0.79 | 0.0026 | 0.80 | 0.83 | 0.0140 |
| 0.81 | 0.79 | 0.0040 | 0.81 | 0.85 | 0.0167 |
| 0.82 | 0.80 | 0.0053 | 0.82 | 0.88 | 0.0171 |
| 0.83 | 0.80 | 0.0065 | 0.83 | 0.90 | 0.0164 |
| 0.84 | 0.80 | 0.0075 | 0.84 | 0.91 | 0.0155 |
| 0.85 | 0.81 | 0.0084 | 0.85 | 0.93 | 0.0144 |
| 0.86 | 0.81 | 0.0093 | 0.86 | 0.94 | 0.0134 |
| 0.87 | 0.82 | 0.0101 | 0.87 | 0.95 | 0.0124 |
| 0.88 | 0.82 | 0.0108 | 0.88 | 0.96 | 0.0116 |
| 0.89 | 0.83 | 0.0114 | 0.89 | 0.97 | 0.0109 |
| 0.90 | 0.83 | 0.0120 | 0.90 | 0.99 | 0.0103 |
| 0.91 | 0.84 | 0.0124 | 0.91 | 1.00 | 0.0098 |
| 0.92 | 0.84 | 0.0127 | 0.92 | 1.00 | 0.0093 |
| 0.93 | 0.85 | 0.0129 | 0.93 | 1.01 | 0.0089 |
| 0.94 | 0.86 | 0.0129 | 0.94 | 1.02 | 0.0086 |
| 0.95 | 0.87 | 0.0128 | 0.95 | 1.03 | 0.0083 |
| 0.96 | 0.88 | 0.0126 | 0.96 | 1.04 | 0.0081 |
| 0.97 | 0.89 | 0.0122 | 0.97 | 1.05 | 0.0079 |
| 0.98 | 0.90 | 0.0118 | 0.98 | 1.06 | 0.0077 |
| 0.99 | 0.90 | 0.0114 | 0.99 | 1.07 | 0.0075 |
| 1.00 | 0.92 | 0.0109 | 1.00 | 1.08 | 0.0074 |
| 1.01 | 0.93 | 0.0104 | 1.01 | 1.09 | 0.0073 |
| 1.02 | 0.94 | 0.0100 | 1.02 | 1.10 | 0.0073 |
| 1.03 | 0.95 | 0.0095 | 1.03 | 1.11 | 0.0074 |
| 1.04 | 0.96 | 0.0091 | 1.04 | 1.12 | 0.0075 |
| 1.05 | 0.97 | 0.0088 | 1.05 | 1.13 | 0.0076 |
| 1.06 | 0.98 | 0.0085 | 1.06 | 1.14 | 0.0079 |
| 1.07 | 0.99 | 0.0082 | 1.07 | 1.15 | 0.0082 |
| 1.08 | 1.00 | 0.0079 | 1.08 | 1.16 | 0.0086 |
| 1.09 | 1.01 | 0.0077 | 1.09 | 1.17 | 0.0091 |
| 1.10 | 1.02 | 0.0076 | 1.10 | 1.18 | 0.0097 |
| 1.11 | 1.03 | 0.0075 | 1.11 | 1.19 | 0.0104 |
| 1.12 | 1.04 | 0.0075 | 1.12 | 1.20 | 0.0112 |
| 1.13 | 1.05 | 0.0075 | 1.13 | 1.21 | 0.0121 |
| 1.14 | 1.06 | 0.0076 | 1.14 | 1.22 | 0.0131 |
| 1.15 | 1.07 | 0.0078 | 1.15 | 1.23 | 0.0143 |
| 1.16 | 1.08 | 0.0081 | 1.16 | 1.25 | 0.0157 |
| 1.17 | 1.09 | 0.0084 | 1.17 | 1.26 | 0.0175 |
| 1.18 | 1.10 | 0.0088 | 1.18 | 1.27 | 0.0201 |
| 1.19 | 1.11 | 0.0092 | 1.19 | 1.28 | 0.0244 |
| 1.20 | 1.12 | 0.0097 | 1.20 | 1.30 | 0.0322 |
| 1.21 | 1.13 | 0.0102 | 1.21 | 1.31 | 0.0400 |
| 1.22 | 1.14 | 0.0108 | 1.22 | 1.33 | 0.0460 |
| 1.23 | 1.15 | 0.0114 | 1.23 | 1.35 | 0.0560 |
| 1.24 | 1.15 | 0.0121 | 1.24 | 1.37 | 0.0678 |
| 1.25 | 1.16 | 0.0128 | 1.25 | 1.39 | 0.0764 |
| 1.26 | 1.17 | 0.0136 | 1.26 | 1.43 | 0.0808 |
| 1.27 | 1.18 | 0.0144 | 1.27 | 1.47 | 0.0808 |
| 1.28 | 1.19 | 0.0154 | 1.28 | 1.50 | 0.0787 |
| 1.29 | 1.19 | 0.0164 | 1.29 | 1.53 | 0.0755 |
| 1.30 | 1.20 | 0.0176 | 1.30 | 1.54 | 0.0717 |
| 1.31 | 1.21 | 0.0189 | 1.31 | 1.55 | 0.0678 |
| 1.32 | 1.21 | 0.0203 | 1.32 | 1.56 | 0.0640 |
| 1.33 | 1.22 | 0.0219 | 1.33 | 1.56 | 0.0605 |
| 1.34 | 1.23 | 0.0237 | 1.34 | 1.56 | 0.0573 |
| 1.35 | 1.23 | 0.0256 | 1.35 | 1.56 | 0.0545 |
| 1.36 | 1.24 | 0.0278 | 1.36 | 1.57 | 0.0521 |
| 1.37 | 1.24 | 0.0302 | 1.37 | 1.57 | 0.0500 |
| 1.38 | 1.24 | 0.0330 | 1.38 | 1.57 | 0.0482 |
| 1.39 | 1.25 | 0.0360 | 1.39 | 1.57 | 0.0467 |
| 1.40 | 1.25 | 0.0395 | 1.40 | 1.57 | 0.0455 |
| 1.41 | 1.25 | 0.0438 | 1.41 | 1.57 | 0.0443 |
| 1.42 | 1.26 | 0.0495 | 1.42 | 1.57 | 0.0434 |
| 1.43 | 1.26 | 0.0580 | 1.43 | 1.57 | 0.0426 |
| 1.44 | 1.26 | 0.0679 | 1.44 | 1.57 | 0.0418 |
| 1.45 | 1.27 | 0.0757 | 1.45 | 1.57 | 0.0411 |
| 1.46 | 1.27 | 0.0813 | 1.46 | 1.57 | 0.0405 |
| 1.47 | 1.27 | 0.0877 | 1.47 | 1.57 | 0.0399 |
| 1.48 | 1.27 | 0.0929 | 1.48 | 1.57 | 0.0393 |
| 1.49 | 1.28 | 0.0981 | 1.49 | 1.57 | 0.0387 |
| 1.50 | 1.28 | 0.1036 | 1.50 | 1.57 | 0.0380 |
| 1.51 | 1.28 | 0.1074 | 1.51 | 1.57 | 0.0372 |
| 1.52 | 1.29 | 0.1101 | 1.52 | 1.57 | 0.0363 |
| 1.53 | 1.29 | 0.1123 | 1.53 | 1.57 | 0.0352 |
| 1.54 | 1.30 | 0.1143 | 1.54 | 1.57 | 0.0337 |
| 1.55 | 1.31 | 0.1161 | 1.55 | 1.57 | 0.0314 |
| 1.56 | 1.33 | 0.1179 | 1.56 | 1.57 | 0.0276 |
| 1.57 | 1.45 | 0.1058 | 1.57 | 1.57 | 0.0187 |

*Note.* SE refers to bootstrapped standard error.
